# Comparative Study of *Helicobacter pylori-*Infected Gastritis in Okinawa and Tokyo Based on the Kyoto Classification of Gastritis

**DOI:** 10.3390/jcm11195739

**Published:** 2022-09-28

**Authors:** Shotaro Oki, Tsutomu Takeda, Mariko Hojo, Ryota Uchida, Nobuyuki Suzuki, Daiki Abe, Atsushi Ikeda, Yoichi Akazawa, Hiroya Ueyama, Shuko Nojiri, Shinichi Hoshino, Hayashi Shokita, Akihito Nagahara

**Affiliations:** 1 Department of Gastroenterology, Juntendo University School of Medicine, 2-1-1 Hongo, Bunkyo-ku, Tokyo 113-8421, Japan; 2 Medical Technology Innovation Center, Juntendo University School of Medicine, 2-1-1 Hongo, Bunkyo-ku, Tokyo 113-8421, Japan; 3 Department of Gastroenterology, Okinawa Prefectural Hokubu Hospital, 2-12-3 Oonaka, Nagoshi 905-8512, Japan; 4 Department of Gastroenterology, Northern Okinawa Medical Center, 1712-3 Umusa, Nagoshi 905-8611, Japan

**Keywords:** *Helicobacter pylori*, Kyoto Classification of Gastritis, *cagA*, Okinawa, endoscopic findings

## Abstract

The incidence of gastric cancer in Okinawa Prefecture is the lowest in Japan, which is attributed to differences in strains of *Helicobacter pylori* in Okinawa and other prefectures in Japan. Our aim was to compare the endoscopic findings of *H. pylori*-infected gastric mucosa in Okinawa and Tokyo. Patients who underwent upper gastrointestinal endoscopy (UGI) at Northern Okinawa Medical Center (Okinawa group) and Juntendo University Hospital (Tokyo group) from April 2019 to March 2020 were included. Patients diagnosed with *H. pylori-*infected gastric mucosa were retrospectively compared between the Okinawa and Tokyo groups according to the Kyoto Classification of Gastritis. The numbers of subjects (Okinawa/Tokyo) were 435/352, male/female ratio was 247:188/181:171, and age was 53.3 ± 14.7/64.6 ± 14.3 (mean ± standard deviation) years. Regarding the Kyoto Classification of Gastritis, the prevalence (Okinawa/Tokyo) of the closed type of atrophic gastritis was 73%/37% (*p* < 0.001), diffuse redness 80%/84% (*p* = 0.145), mucosal swelling 46%/46% (*p* = 0.991), enlarged fold 26%/32% (*p* = 0.048), spotty redness 77%/68% (*p* = 0.002), sticky mucus 17%/36% (*p* < 0.001), and intestinal metaplasia 32%/42% (*p* < 0.001). Age analysis also revealed that closed-type atrophy and spotty redness were more frequent in the Okinawa group than in the Tokyo group. There may be regional differences in endoscopic findings of *H. pylori*-infected gastric mucosa between Okinawa and Tokyo.

## 1. Introduction

*Helicobacter pylori* (*H. pylori*) infection is known to be an important pathogenic factor for gastric cancer [1,2,3,4]. The incidence of gastric cancer is generally higher in East Asia, while it is lower in North America, Northern Europe, and Africa, suggesting regional differences throughout the world [5]. The prevalence of *H. pylori* in Okinawa is not significantly different from that in other regions of Japan [1,6,7]. However, the age-standardized incidence rate of gastric cancer in Japan is 43.1%, while the rate in Okinawa is 20.4%, the lowest age-standardized incidence rate among prefectures in Japan [8]. Even within Japan, regional differences are recognized. This may be due to differences in the cagA gene of *H. pylori* [9,10,11,12]. Regional differences and polymorphisms in *H. pylori* genotypes also differ in their influence as virulence factors, which has been studied in recent years as one of the factors contributing to regional differences in gastric cancer incidence.

In addition, in routine endoscopic practice, the risk of gastric cancer is approximately 20 times higher in *H.* *pylori*-infected patients than in *H.* *pylori*-uninfected patients [13], and it is important to determine the *H. pylori* infection status from the background mucosa. Therefore, in 2014, the Kyoto Classification of Gastritis was published to systematically summarize the findings of *H. pylori*-associated gastritis for the first time [14]. The English version was published in 2017, and the second edition with new findings was published in 2018 [15,16]. Many Japanese studies have evaluated the Kyoto Classification of Gastritis, but recently some reports have confirmed its usefulness in European countries [17]. The Kyoto Classification of Gastritis allows efficient evaluation of gastric cancer risk based on the state of the background mucosa with 19 endoscopic findings. In particular, diffuse redness, mucosal swelling, enlarged fold, sticky mucus, and spotty redness are considered findings suggestive of *H. pylori* infection [14,15,16,18,19,20,21,22].

Although differences in gastric cancer incidence rates and strains of *H. pylori* are observed between Okinawa and other regions of Japan, there have been no reports comparing gastritis findings in Okinawa with those in other regions of Japan. The purpose of this study was to clarify the differences and characteristics of endoscopic findings of *H. pylori*-infected gastric mucosa between Okinawa and Tokyo on the mainland of Japan.

## 2. Materials and Methods

### 2.1. Patients

Patients who underwent upper gastrointestinal endoscopy (UGI) at Northern Okinawa Medical Center (Okinawa Group) and Juntendo University Hospital (Tokyo Group) from April 2019 to March 2020 were included in this retrospective study. Inclusion criteria were patients who were 18 years old or older, and who were infected with *H.* *pylori* at the time of UGI. Patients were considered to be infected with *H. pylori* when at least one of the urea breath tests with cutoff value of 2.5 per 1000, serum *H. pylori* antibody test with cutoff value of 10 U/mL (E-plate; Eiken Chemical, Tokyo, Japan), and stool *H. pylori* antigen test was positive from April 2017 to March 2020.

Exclusion criteria were patients who had undergone gastrectomy, those whose mucosa was poorly observed due to food residues, those who did not have pure *H.* *pylori*-infected gastric mucosa due to coexistence of liver cirrhosis and/or autoimmune gastritis, and those who had undergone *H. pylori* eradication. This retrospective study was conducted according to the guidelines of the Helsinki Declaration and was approved by the Ethics Committee of Northern Okinawa Medical Center (2019-5) and the Ethics Committee of Juntendo University Hospital (E21-0335-H01). Patient consent was waived because the design of this study was a retrospective clinical documentation study.

### 2.2. Methods

This retrospective cross-sectional study investigated the endoscopic findings of patients with *H. pylori*-infected gastric mucosa according to the Kyoto Classification of Gastritis in Okinawa and Tokyo. Six expert endoscopists in Tokyo and two expert endoscopists in Okinawa who each have performed more than 1500 UGIs, independently and retrospectively reviewed all photographs of endoscopic examinations performed at the Okinawa and Tokyo hospitals during the study period, and evaluated the endoscopic findings in the photographs according to the Kyoto Classification of Gastritis [14,15,16]. Regarding atrophy, the Kimura-Takemoto classification [23] was used to evaluate the degree of spread of gastric mucosal atrophy. C-1, C-2, and C-3 were classified as the closed type of atrophy, and O-1, O-2, and O-3 as the open type.

### 2.3. Statistical Analysis

All data on patients’ background characteristics and endoscopic findings according to the Kyoto Classification of Gastritis in each group were expressed as mean and standard deviation (SD) for continuous variables and as the number (percentage) for categorical variables. The prevalence of each characteristic was examined using the *t*-test, chi-square test or Fisher’s exact test, with *p* < 0.05 being considered statistically significant. In addition, the number of gastric cancer cases in patients over 65 years of age has been increasing in Japan [8]. Therefore, in order to analyze the risk of gastric cancer and age changes, we conducted subgroup analyses in patients under 65 years of age and in patients 65 years of age or older. We compared the distribution of endoscopic features in the Kyoto Classification of Gastritis between the patients in Tokyo and patients in Okinawa using the logistic regression model adjusting for age and sex. All analyses were conducted using SAS software (SAS Institute, Cary, NC, USA). Odds ratio (OR) value was presented with the 95% confidence interval (CI). All tests were two-sided and statistical significance was set at *p* < 0.05.

## 3. Results

### 3.1. Patients Studied

**Figure 1:**
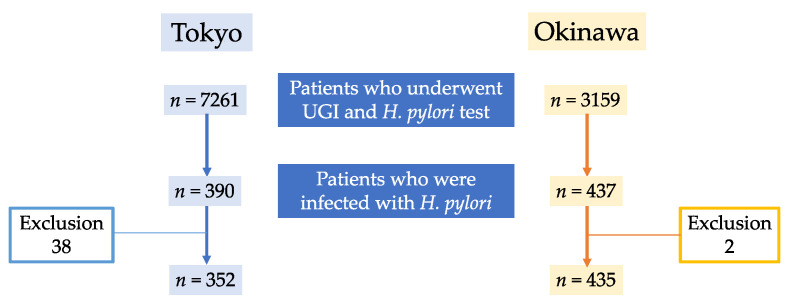
Flow diagram of the patients.

Patient flow is shown in Figure 1. During the study period, 7261 patients at the hospital in Tokyo and 3159 patients at the hospital in Okinawa underwent UGI and had been tested for *H. pylori*. Of these, 390 patients were 18 years of age or older and infected with *H. pylori* at the hospital in Tokyo, and 437 patients were 18 years of age or older and infected with *H. pylori* at the hospital in Okinawa. Based on the exclusion criteria, 38 cases at the hospital in Tokyo (6 patients after gastrectomy, 8 patients with liver cirrhosis, 11 foreigners, 1 patient with autoimmune gastritis, 3 patients with insufficient observation of the gastric mucosa, and 9 patients after *H. pylori* eradication) and 2 cases at the hospital in Okinawa (2 patients with liver cirrhosis) were excluded. Therefore, the Tokyo group included 352 cases and the Okinawa group included 435 cases. The mean (± standard deviation) age of the 352 patients in the Tokyo group and the 435 patients in the Okinawa group was 64.6 ± 14.3 and 53.3 ± 14.7, respectively (*p* < 0.001). There was no difference in sex distribution (male/female) between the Tokyo group (181/171) and the Okinawa group (247/188) (*p* = 0.133) (Table 1). The numbers of patients under 65 years old (Tokyo/Okinawa group) were 156/327 patients and the numbers of patients 65 years old or older (Tokyo/Okinawa group) were 196/108 patients (Table 2).

### 3.2. Endoscopic Findings of H. pylori Gastritis Based on the Kyoto Classification of Gastritis

**Figure 2:**
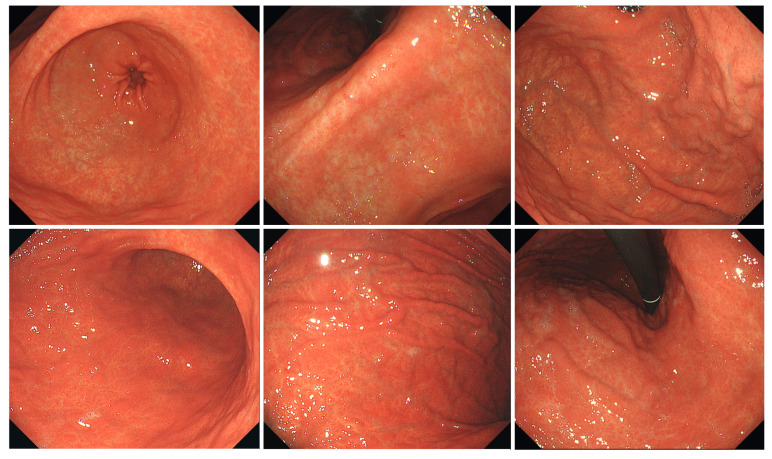
Typical endoscopic images of *H. pylori*-infected gastric mucosa in the Tokyo group. The patient is a 75-year-old woman. O-1 atrophy is observed, with diffuse redness, spotty redness, and mucosal swelling. Overall, it has a reddish tint.

**Figure 3:**
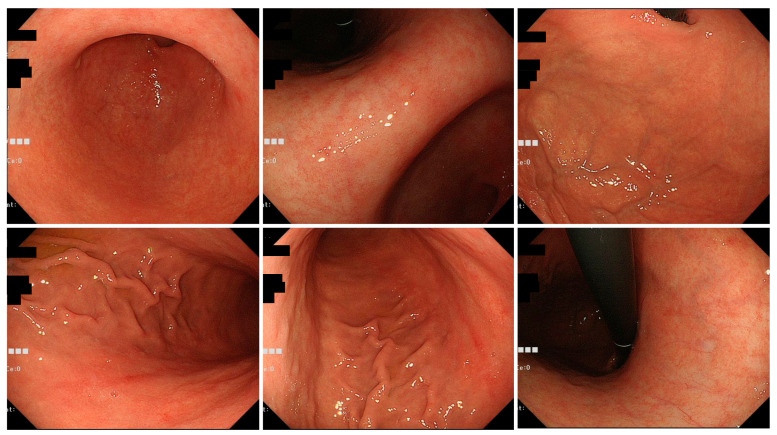
Typical endoscopic images of *H. pylori*-infected gastric mucosa in the Okinawa group. This patient is an 83-year-old woman. O-2 atrophy is observed, and there are only a few findings of *H. pylori* infection. Mild spotty redness on the posterior wall of the gastric body is observed.

**Figure 4:**
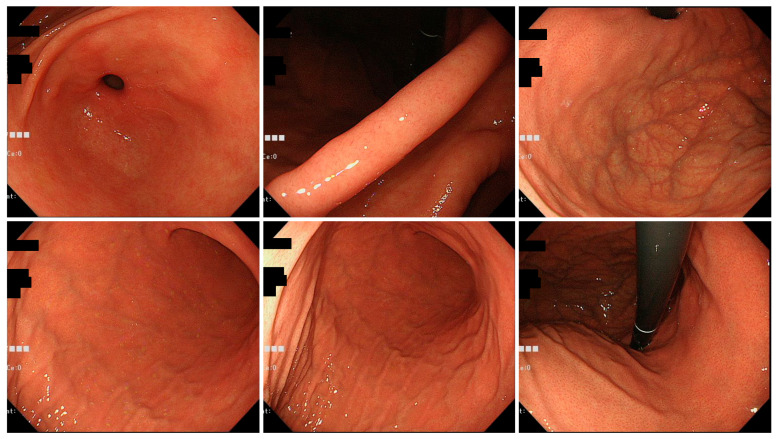
Typical endoscopic images of *H. pylori*-infected gastric mucosa in the Okinawa group. This patient is a 46-year-old woman. C-1 atrophy is observed, and there are few findings of *H. pylori* infection.

Typical endoscopic findings in the Tokyo and Okinawa groups are shown in Figure 2, Figure 3 and Figure 4.

Closed-type atrophy was more common in the Okinawa group than in the Tokyo group [318/435 (73.1%) vs. 131/352 (37.2%)] (*p* < 0.001). Diffuse redness, mucosal swelling, enlarged fold, spotty redness, and sticky mucus are the findings of *H. pylori-*infected gastric mucosa. The following characteristics were significantly more common in the Tokyo group than in the Okinawa group: enlarged fold [114/352 (32.4%) vs. 113/435 (26.0%), *p* = 0.048], sticky mucus [125/352 (35.5%) vs. 73/435 (16.8%), *p* < 0.001], foveolar-hyperplastic polyp [50/352 (14.2%) vs. 39/435 (8.97%), *p* = 0.021], intestinal metaplasia [147/352 (41.8%) vs. 142/435 (32.6%), *p* = 0.007], and xanthoma [33/352 (9.38%) vs. 20/435 (4.60%), *p* < 0.001]. The following characteristics were significantly less common in the Tokyo group than in the Okinawa group: spotty redness [239/352 (67.9%) vs. 338/435 (77.7%), *p* = 0.002], patchy redness [29/352 (8.24%) vs. 59/435 (13.6%), *p* = 0.018], and hematin [6/352 (1.70%) vs. 28/435 (6.44%), *p* = 0.001] (Table 3).

### 3.3. Age Analysis

Among patients with *H. pylori*-infected gastric mucosa under 65 years of age, closed-type atrophy was more common in the Okinawa group than in the Tokyo group [270/327 (82.6%) vs. 82/156 (52.6%), *p* < 0.001]. Similar to the results of the main analysis, enlarged fold [51/156 (32.7%) vs. 76/327(23.2%), Tokyo group vs. Okinawa group, *p* = 0.027], sticky mucus [58/156 (37.2%) vs. 45/327 (13.7%), *p* < 0.001], foveolar-hyperplastic polyp [21/156 (13.5%) vs. 23/327 (7.03%), *p* = 0.021], and xanthoma [9/156 (5.77%) vs. 7/327 (2.14%), *p* = 0.037] were more common in the Tokyo group. Spotty redness [100/156 (64.1%) vs. 248/327 (75.8%), Tokyo group vs. Okinawa group, *p* = 0.007], patchy redness [7/156 (4.48%) vs. 39/327 (11.9%), *p* = 0.009], and hematin [2/156 (1.28%) vs. 23/327 (7.03%), *p* = 0.008] were less common in the Tokyo group than in the Okinawa group (Table 4).

Even among patients aged 65 years or older, closed-type atrophy was more frequent in the Okinawa group than in the Tokyo group [48/108 (44.4%) vs. 49/196 (25.0%), *p* < 0.001]. In terms of *H. pylori-*infected gastric mucosa findings, spotty redness was less common in the Tokyo group than in the Okinawa group [139/196 (70.9%) vs. 90/108 (83.3%), *p* = 0.016]. The frequencies of other findings of *H. pylori-*infected gastric mucosa were not significantly different between the Tokyo and Okinawa groups (Table 4).

### 3.4. Age-Adjusted Analysis

The data were age-adjusted as the Okinawa group was significantly younger than the Tokyo group. In the Okinawa group, closed-type atrophy (closed:1, open:0) (OR [95%Cl] = 2.843 [2.032–3.985], *p* < 0.001), spotty redness (OR [95%Cl] = 1.947 [1.379–2.761], *p* < 0.001), patchy redness (OR [95%Cl] = 2.336 [1.421–3.917], *p* = 0.001), and hematin (OR [95%Cl] = 3.526 [1.471–9.849], *p* = 0.008) were more common and sticky mucus was less common (OR [95%Cl] = 0.439 [0.306–0.626], *p* < 0.001) (Table 5).

## 4. Discussion

Comparison of the endoscopic findings of the gastric mucosa of *H. pylori*-infected patients according to the Kyoto Classification of Gastritis suggested that there may be regional differences in endoscopic findings of *H.* *pylori*-infected mucosa between the Okinawa and Tokyo groups. In addition, spotty redness in the Kyoto Classification of Gastritis was useful for the endoscopic diagnosis of *H. pylori* infection in the Okinawa group. To our knowledge, this is the first study to compare regional differences in endoscopic findings of *H. pylori*-infected gastric mucosa according to the Kyoto Classification of Gastritis.

In the Kyoto Classification of Gastritis, atrophy, intestinal metaplasia, diffuse redness, and nodularity are risk factors for gastric cancer [22,24,25,26,27,28]. Enlarged fold has also been weakly associated with undifferentiated gastric carcinoma [26]. Regarding the inhibitory effect of *H. pylori* eradication on gastric carcinogenesis, the greater the extent of mucosal atrophy, the lower the inhibitory effect of eradication on carcinogenesis [29]. In the present study, the Okinawa group had a higher frequency of closed-type atrophy than the Tokyo group, and lower frequencies of enlarged fold and intestinal metaplasia, suggesting that the risk of gastric cancer according to the Kyoto Classification of Gastritis was low in Okinawa.

The prevalence of diffuse redness was not significantly different between the two groups, but was less common in the Okinawa group. In addition, the degree of atrophy was weaker in the Okinawa group, and the presence of *H. pylori* infection findings such as enlarged fold and sticky mucus was less conspicuous, making the endoscopic diagnosis of *H. pylori*-infected gastric mucosa more difficult than in the Tokyo group. The Okinawa group showed predominantly more spotty redness as a finding of *H. pylori*-infected gastric mucosa. It is considered that diffuse redness was less noticeable in the Okinawa group than in the Tokyo group, and therefore, it was inferred that the spotty redness was relatively easy to see endoscopically in the Okinawa group. Spotty redness was considered to be helpful in the diagnosis of *H. pylori*-infected gastric mucosa among the endoscopic findings in Okinawa.

Diffuse redness is a basic finding of *H. pylori* infection as well as mucosal swelling, and correlates predominantly with the degree of neutrophilic and mononuclear cell infiltration caused by *H. pylori* infection [19]. The diffuse redness in the Okinawa group was weak and difficult to diagnose endoscopically, which means that the *H. pylori* strains in Okinawa are less inflammatory. In addition, more hematin adherence was observed in the Okinawa group than in the Tokyo group. Hematin is considered to appear when the intragastric pH is highly acidic [30], and it has been reported that *H. pylori*-uninfected individuals have a higher acid secretory capacity than *H. pylori*-infected individuals [31,32,33]. In the age-adjusted analysis, the Okinawa group had significantly fewer cases of sticky mucus, foveolar hyperplastic polyp, and enlarged fold in those under 65 years of age, but in those 65 years of age and older, these frequencies increased to the same levels as those in the Tokyo group, and the differences were no longer significant. This suggests that the inflammation caused by *H. pylori* was stronger in the Tokyo group and weaker in the Okinawa group, and that the mucosal findings were therefore less noticeable in the younger age group in Okinawa. On the other hand, the elderly group showed changes due to long-term inflammation, and the difference in mucosal findings between the Tokyo group and the Okinawa group became smaller, although not regarding the extent of atrophy.

Okinawa is an island far from the mainland of Japan, and one of the reasons for the low incidence of gastric cancer in Okinawa is thought to be the different environmental factors and dietary habits compared to those in the rest of Japan [34]. Furthermore, genetic analysis of *H. pylori* has pointed to differences in *H. pylori* strains in Okinawa compared to those in other regions of Japan as a cause for the different incidence of gastric cancer [9,10,11,12]. The *cagA* gene is known to be a representative pathogenic factor for gastric cancer in *H. pylori,* but the CagA protein itself is not essential for the survival of the bacteria, and there are *cagA*-positive and *cagA*-negative strains. *cagA*-positive strains are more pathogenic than *cagA*-negative strains and are reported to increase the risk of peptic ulcers and gastric cancer [35,36]. *cagA*-negative strains are more common in South Africa, with a higher proportion of *cagA*-positive strains in the Asian region. Among *cagA* strains, East Asian-type *cagA* strains are considered to be more virulent compared to Western-type *cagA* strains [37]. In Japan, almost 100% of the *H. pylori* strains are East Asian-type *cagA* strains, except in Okinawa. In Okinawa, about 15% of *H. pylori* strains are *cagA*-negative and about 15% of strains are Western-type *cagA* strains, and this difference in *H. pylori* strains is thought to be the reason that the gastric cancer incidence rate is lowest in Okinawa Prefecture among prefectures in Japan [9,10,11,12].

About 50% of the world’s population is a carrier of *H. pylori*. Because *H. pylori* is transmitted mainly by vertical transmission from parent to child and has a high mutation rate compared to human genes, recent studies have shown that it is possible to estimate not only the diversity of its pathogenicity but also the history of human migration by studying its genotypes in various regions of the world [12,38,39,40]. Genetic analysis of *H. pylori* by the multi locus sequence typing (MLST) method has demonstrated the existence of two new types unique to Okinawa, not found in other regions, one of which diverged from other strains tens of thousands of years ago [12]. It has been speculated that there were multiple waves of human migration starting in Africa [12,38,39,40,41,42], and the Okinawa-specific *H. pylori* strains are thought to be the result of early migratory people. At present, the route of arrival of the new type of *H. pylori* strains recognized in Okinawa is unknown, but Okinawa has its own strains of *H. pylori,* and it is possible that regional differences in gastritis findings were observed due to differences in these *H. pylori* strains.

There are several limitations in this study. Because this was a retrospective cross-sectional study and *H. pylori* gene analysis was not performed, the *H. pylori* strains could not be identified. In addition, each endoscopist evaluated the endoscopic findings independently, which may have resulted in interobserver variability. The methods used to diagnose *H. pylori* infection were different in each group, and the accuracy, sensitivity, and specificity of each *H. pylori* test varied, which may have affected the results of this study. The present study did not consider the effects of drugs that alter the gastric mucosa and duodeno-gastric reflux, and age stratification analysis was not performed. As the number of hospitals surveyed in this study was limited, we have not been able to examine whether the study is representative of the general population in Japan. Among patients who underwent UGI, the Okinawa group underwent endoscopy based on medical checkup, whereas the Tokyo group underwent endoscopy based on disease, suggesting selection bias. Therefore, a prospective study considering other factors that may affect the gastric mucosa and combining *H. pylori* gene analysis and age stratification analysis will be required to confirm our findings.

## 5. Conclusions

When the endoscopic findings according to the Kyoto Classification of Gastritis were compared between the Okinawa and Tokyo groups, the Okinawa group showed a higher percentage of closed-type atrophy and a difference in inflammation-related findings of *H. pylori*-infected gastric mucosa compared with the Tokyo group. Among the endoscopic features in the Kyoto Classification of Gastritis, spotty redness was considered to be useful for the diagnosis of *H. pylori* infection in the Okinawa group. Our results suggested the possibility of regional differences in endoscopic findings of *H.* *pylori*-infected gastric mucosa between Okinawa and Tokyo.

## Figures and Tables

**Table 1:** Baseline characteristics.

		Tokyo Group	Okinawa Group	*p*-Value
Number of patients		352	435	
Age (yr; mean ± SD)		64.6 ± 14.3	53.3 ± 14.7	<0.001
Sex	male	181	247	0.133
	female	171	188	

SD, standard deviation.

**Table 2:** Baseline characteristics of the patients under 65 years old and patients 65 years old or older.

		Tokyo Group	Okinawa Group	*p*-Value
Group of patients under 65 years old	Number of patients	156	327	
	Age (yr; mean ± SD)	52.0 ± 11.3	46.9 ± 10.5	<0.001
	Male/female	72/84	181/146	0.058
Group of patients 65 years old or older	Number of patients	196	108	
	Age (yr; mean ± SD)	74.6 ± 6.1	72.7 ± 5.6	0.006
	Male/female	109/87	66/42	0.353

**Table 3:** Comparison of endoscopic findings according to the Kyoto Classification of Gastritis between the Tokyo and Okinawa groups.

Kyoto Classification of Gastritis		Tokyo Group (%)	Okinawa Group (%)	*p-*Value
		*n* = 352	*n* = 435	
Atrophy ^1^	closed	131 (37.2) ^2^	318 (73.1)	<0.001
	open	221 (62.8)	117 (26.9)	
diffuse redness		298 (84.7)	351 (80.7)	0.145
mucous swelling		165 (46.9)	212 (48.7)	0.696
enlarged fold		114 (32.4)	113 (26.0)	0.048
spotty redness		239 (67.9)	338 (77.7)	0.002
sticky mucus		125 (35.5)	73 (16.8)	<0.001
foveolar-hyperplastic polyp		50 (14.2)	39 (8.97)	0.021
patchy redness		29 (8.24)	59 (13.6)	0.018
intestinal metaplasia		147 (41.8)	142 (32.6)	0.007
nodularity		23 (6.53)	37 (8.51)	0.300
xanthoma		33 (9.38)	20 (4.60)	0.007
hematin		6 (1.70)	28 (6.44)	0.001

^1^ Kimura-Takemoto classification. ^2^ Data are expressed as the number of patients (%).

**Table 4:** Subgroup analyses in each age group below and above 65 years of age between the Tokyo and Okinawa patients. (**a**) The under-65-years age group; (**b**) the 65-years-and-older age group.

(**a**) The under-65-years age group				
**Kyoto Classification of Gastritis**		**Tokyo Group (%)**	**Okinawa Group (%)**	***p*-Value**
		***n* = 156**	***n* = 327**	
Atrophy	closed	82 (52.6)	270 (82.6)	<0.001
	open	74 (47.4)	57 (17.4)	
diffuse redness		132 (84.6)	260 (79.6)	0.180
mucosal swelling		71 (45.5)	149 (45.6)	0.991
enlarged fold		51 (32.7)	76 (23.2)	0.027
spotty redness		100 (64.1)	248 (75.8)	0.007
sticky mucus		58 (37.2)	45 (13.7)	<0.001
foveolar-hyperplastic polyp		21 (13.5)	23 (7.03)	0.021
patchy redness		7 (4.48)	39 (11.9)	0.009
intestinal metaplasia		48 (30.8)	89 (27.2)	0.340
nodularity		19 (12.2)	36 (11.0)	0.705
xanthoma		9 (5.77)	7 (2.14)	0.037
hematin		2 (1.28)	23 (7.03)	0.008
(**b**) The 65-years-and-older age group				
**Kyoto Classification of Gastritis**		**Tokyo Group (%)**	**Okinawa Group (%)**	***p*-Value**
		***n* = 196**	***n* = 108**	
Atrophy	closed	49 (25.0)	48 (44.4)	<0.001
	open	147 (75.0)	60 (55.6)	
diffuse redness		166 (84.7)	91 (84.3)	0.920
mucosal swelling		94 (48.0)	63 (58.3)	0.083
enlarged fold		63 (32.1)	37 (34.3)	0.707
spotty redness		139 (70.9)	90 (83.3)	0.016
sticky mucus		67 (34.2)	28 (26.0)	0.137
foveolar-hyperplastic polyp		29 (14.8)	16 (14.8)	0.966
patchy redness		22 (11.2)	20 (18.5)	0.078
intestinal metaplasia		99 (50.5)	53 (49.1)	0.811
nodularity		4 (2.04)	1 (0.93)	0.659
xanthoma		24 (12.2)	13 (12.0)	0.958
hematin		4 (2.04)	5 (4.63)	0.288

**Table 5:** Odds ratios of each endoscopic feature in the Kyoto Classification of Gastritis between the Tokyo and Okinawa groups in logistic regression analysis.

Kyoto Classification of Gastritis	OR *	95%CI	*p*-Value
atrophy (closed 1; open 0)	2.843	2.032–3.985	<0.001
diffuse redness	0.787	0.524–1.174	0.244
mucosal swelling	1.221	0.900–1.658	0.200
enlarged fold	0.816	0.583–1.141	0.234
spotty redness	1.947	1.379–2.761	<0.001
sticky mucus	0.439	0.306–0.626	<0.001
foveolar hyperplastic polyp	0.816	0.504–1.317	0.406
patchy redness	2.336	1.421–3.917	0.001
intestinal metaplasia	0.975	0.708–1.346	0.876
nodularity	0.601	0.318–1.142	0.117
xanthoma	0.731	0.390–1.344	0.319
hematin	3.526	1.471–9.849	0.008

* OR: Odds Ratio (reference: Tokyo prefecture) adjusting for age and sex.

## Data Availability

All data supporting this study are available in the article.

## References

[1] Warren J.R., Marshall B. (1983). Unidentified curved bacilli on gastric epithelium in active chronic gastritis. Lancet.

[2] (1994). NIH Consensus Conference. *Helicobacter pylori* in peptic ulcer disease. NIH Consensus Development Panel on *Helicobacter pylori* in Peptic Ulcer Disease. JAMA.

[3] Marshall B.J., Goodwin C.S., Warren J.R., Murray R., Blincow E.D., Blackbourn S.J., Phillips M., Waters T.E., Sanderson C.R. (1988). Prospective double-blind trial of duodenal ulcer relapse after eradication of *Campylobacter pylori*. Lancet.

[4] Parsonnet J., Friedman G.D., Vandersteen D.P., Chang Y., Vogelman J.H., Orentreich N., Sibley R.K. (1991). *Helicobacter pylori* infection and the risk of gastric carcinoma. N. Engl. J. Med..

[5] Bray F., Ferlay J., Soerjomataram I., Siegel R.L., Torre L.A., Jemal A. (2018). Global cancer statistics 2018: GLOBOCAN estimates of incidence and mortality worldwide for 36 cancers in 185 countries. CA Cancer J. Clin..

[6] Ito S., Azuma T., Murakita H., Hirai M., Miyaji H., Ito Y., Ohtaki Y., Yamazaki Y., Kuriyama M., Keida Y. (1996). Profile of *Helicobacter pylori* cytotoxin derived from two areas of Japan with different prevalence of atrophic gastritis. Gut.

[7] Jones K.R., Joo Y.M., Jang S., Yoo Y.J., Lee H.S., Chung I.S., Olsen C.H., Whitmire J.M., Merrell D.S., Cha J.H. (2009). Polymorphism in the CagA EPIYA motif impacts development of gastric cancer. J. Clin. Microbiol..

[8] (2018). Cancer Statistics. Cancer Information Service, National Cancer Center, Japan (National Cancer Registry, Ministry of Health, Labour and Welfare). https://ganjoho.jp/reg_stat/statistics/data/dl/en.html.

[9] Azuma T., Yamakawa A., Yamazaki S., Ohtani M., Ito Y., Muramatsu A., Suto H., Yamazaki Y., Keida Y., Higashi H. (2004). Distinct diversity of the cag pathogenicity island among *Helicobacter pylori* strains in Japan. J. Clin. Microbiol..

[10] Satomi S., Yamakawa A., Matsunaga S., Masaki R., Inagaki T., Okuda T., Suto H., Ito Y., Yamazaki Y., Kuriyama M. (2006). Relationship between the diversity of the cagA gene of *Helicobacter pylori* and gastric cancer in Okinawa, Japan. J. Gastroenterol..

[11] Yamazaki S., Yamakawa A., Okuda T., Ohtani M., Suto H., Ito Y., Yamazaki Y., Keida Y., Higashi H., Hatakeyama M. (2005). Distinct diversity of vacA, cagA, and cagE genes of *Helicobacter pylori* associated with peptic ulcer in Japan. J. Clin. Microbiol..

[12] Matsunari O., Shiota S., Suzuki R., Watada M., Kinjo N., Murakami K., Fujioka T., Kinjo F., Yamaoka Y. (2012). Association between *Helicobacter pylori* virulence factors and gastroduodenal diseases in Okinawa, Japan. J. Clin. Microbiol..

[13] Ekström A.M., Held M., Hansson L.E., Engstrand L., Nyrén O. (2001). *Helicobacter pylori* in gastric cancer established by CagA immunoblot as a marker of past infection. Gastroenterology.

[14] Kato M., Inoue K., Murakami K., Kamada T., Haruma K. (2014). Kyoto Classification of Gastritis.

[15] Kato M., Inoue K., Murakami K., Kamada T., Haruma K. (2017). Kyoto Classification of Gastritis.

[16] Kato M., Inoue K., Murakami K., Kamada T., Haruma K. (2018). Kyoto Classification of Gastritis.

[17] Ebigbo A., Marienhagen J., Messmann H. (2021). Regular arrangement of collecting venules and the Kimura-Takemoto classification for the endoscopic diagnosis of *Helicobacter pylori* infection: Evaluation in a Western setting. Dig. Endosc..

[18] Glover B., Teare J., Ashrafian H., Patel N. (2020). The endoscopic predictors of *Helicobacter pylori* status: A meta-analysis of diagnostic performance. Ther. Adv. Gastrointest. Endosc..

[19] Nomura S., Terao S., Adachi K., Kato T., Ida K., Watanabe H., Shimbo T. (2013). Endoscopic diagnosis of gastric mucosal activity and inflammation. Dig. Endosc..

[20] Kato T., Yagi N., Kamada T., Shimbo T., Watanabe H., Ida K. (2013). Diagnosis of *Helicobacter pylori* infection in gastric mucosa by endoscopic features: A multicenter prospective study. Dig. Endosc..

[21] Kato M., Terao S., Adachi K., Nakajima S., Ando T., Yoshida N., Uedo N., Murakami K., Ohara S., Ito M. (2013). Changes in endoscopic findings of gastritis after cure of *H. pylori* infection: Multicenter prospective trial. Dig. Endosc..

[22] Nagahara A., Shiotani A., Iijima K., Kamada T., Fujiwara Y., Kasugai K., Kato M., Higuchi K. (2022). The role of advanced endoscopy in the management of inflammatory digestive diseases (upper gastrointestinal tract). Dig. Endosc..

[23] Kimura K., Takemoto T. (1969). An endoscopic recognition of atrophic border and its significance in chronic gastritis. Endocopy.

[24] Ohno A., Miyoshi J., Kato A., Miyamoto N., Yatagai T., Hada Y., Kusuhara M., Jimbo Y., Ida Y., Tokunaga K. (2020). Endoscopic severe mucosal atrophy indicates the presence of gastric cancer after *Helicobacter pylori* eradication -analysis based on the Kyoto classification. BMC Gastroenterol..

[25] Sakitani K., Nishizawa T., Toyoshima A., Yoshida S., Matsuno T., Yamada T., Irokawa M., Takahashi Y., Nakai Y., Toyoshima O. (2020). Kyoto classification in patients who developed multiple gastric carcinomas after *Helicobacter pylori* eradication. World J. Gastrointest. Endosc..

[26] Shichijo S., Hirata Y., Niikura R., Hayakawa Y., Yamada A., Koike K. (2017). Association between gastric cancer and the Kyoto classification of gastritis. J. Gastroenterol. Hepatol..

[27] Sugimoto M., Ban H., Ichikawa H., Sahara S., Otsuka T., Inatomi O., Bamba S., Furuta T., Andoh A. (2017). Efficacy of the Kyoto Classification of Gastritis in Identifying Patients at High Risk for Gastric Cancer. Intern. Med..

[28] Majima A., Dohi O., Takayama S., Hirose R., Inoue K., Yoshida N., Kamada K., Uchiyama K., Ishikawa T., Takagi T. (2019). Linked color imaging identifies important risk factors associated with gastric cancer after successful eradication of *Helicobacter pylori*. Gastrointest. Endosc..

[29] Take S., Mizuno M., Ishiki K., Yoshida T., Ohara N., Yokota K., Oguma K., Okada H., Yamamoto K. (2011). The long-term risk of gastric cancer after the successful eradication of *Helicobacter pylori*. J. Gastroenterol..

[30] Hatta W., Iijima K., Koike T., Kondo Y., Ara N., Asanuma K., Uno K., Asano N., Imatani A., Shimosegawa T. (2015). Endoscopic findings for predicting gastric acid secretion status. Dig. Endosc..

[31] Haruma K., Kamada T., Kawaguchi H., Okamoto S., Yoshihara M., Sumii K., Inoue M., Kishimoto S., Kajiyama G., Miyoshi A. (2000). Effect of age and *Helicobacter pylori* infection on gastric acid secretion. J. Gastroenterol. Hepatol..

[32] Haruma K., Mihara M., Okamoto E., Kusunoki H., Hananoki M., Tanaka S., Yoshihara M., Sumii K., Kajiyama G. (1999). Eradication of *Helicobacter pylori* increases gastric acidity in patients with atrophic gastritis of the corpus-evaluation of 24-h pH monitoring. Aliment Pharm. Ther..

[33] Koike T., Ohara S., Sekine H., Iijima K., Kato K., Toyota T., Shimosegawa T. (2001). Increased gastric acid secretion after *Helicobacter pylori* eradication may be a factor for developing reflux oesophagitis. Aliment. Pharm. Ther..

[34] Willcox D.C., Willcox B.J., Todoriki H., Suzuki M. (2009). The Okinawan diet: Health implications of a low-calorie, nutrient-dense, antioxidant-rich dietary pattern low in glycemic load. J. Am. Coll. Nutr..

[35] Parsonnet J., Friedman G.D., Orentreich N., Vogelman H. (1997). Risk for gastric cancer in people with CagA positive or CagA negative *Helicobacter pylori* infection. Gut.

[36] Yamaoka Y., Kikuchi S., el-Zimaity H.M., Gutierrez O., Osato M.S., Graham D.Y. (2002). Importance of *Helicobacter pylori* oipA in clinical presentation, gastric inflammation, and mucosal interleukin 8 production. Gastroenterology.

[37] Yamaoka Y. (2010). Mechanisms of disease: *Helicobacter pylori* virulence factors. Nat. Rev. Gastroenterol. Hepatol..

[38] Falush D., Wirth T., Linz B., Pritchard J.K., Stephens M., Kidd M., Blaser M.J., Graham D.Y., Vacher S., Perez-Perez G.I. (2003). Traces of human migrations in *Helicobacter pylori* populations. Science.

[39] Linz B., Balloux F., Moodley Y., Manica A., Liu H., Roumagnac P., Falush D., Stamer C., Prugnolle F., van der Merwe S.W. (2007). An African origin for the intimate association between humans and *Helicobacter pylori*. Nature.

[40] Moodley Y., Linz B., Yamaoka Y., Windsor H.M., Breurec S., Wu J.Y., Maady A., Bernhöft S., Thiberge J.M., Phuanukoonnon S. (2009). The peopling of the Pacific from a bacterial perspective. Science.

[41] Yamaoka Y., Orito E., Mizokami M., Gutierrez O., Saitou N., Kodama T., Osato M.S., Kim J.G., Ramirez F.C., Mahachai V. (2002). *Helicobacter pylori* in North and South America before Columbus. FEBS Lett..

[42] Kersulyte D., Mukhopadhyay A.K., Velapatiño B., Su W., Pan Z., Garcia C., Hernandez V., Valdez Y., Mistry R.S., Gilman R.H. (2000). Differences in genotypes of *Helicobacter pylori* from different human populations. J. Bacteriol..

